# Use as directed? A comparison of software tools intended to check rigor and transparency of published work

**DOI:** 10.1371/journal.pone.0342225

**Published:** 2026-02-13

**Authors:** Peter Eckmann, Adrian Barnett, Alexandra Bannach-Brown, Elisa Pilar Bascunan Atria, Guillaume Cabanac, Louise Delwen Owen Franzen, Małgorzata Anna Gazda, Kaitlyn Hair, James Howison, Halil Kilicoglu, Cyril Labbe, Sarah McCann, Vladislav Nachev, Martijn Roelandse, Maia Salholz-Hillel, Robert Schulz, Gerben ter Riet, Colby Vorland, Anita Bandrowski, Tracey Weissgerber

**Affiliations:** 1 Department of Computer Science and Engineering, UC San Diego, La Jolla, California, United States of America; 2 School of Public Health and Social Work, Queensland University of Technology, Kelvin Grove, Queensland, Australia; 3 QUEST Center for Responsible Research, Berlin Institute of Health at Charité Universitätsmedizin Berlin, Berlin, Germany; 4 Université de Toulouse & Institut Universitaire de France, Toulouse, France; 5 Department of Biological Sciences, University of Montréal, Montréal, Québec, Canada; 6 UCL Social Research Institute, University College London, London, United Kingdom; 7 Information School of the University of Texas at Austin, Austin, Texas, United States of America; 8 School of Information Sciences, University of Illinois Urbana-Champaign, Illinois, United States of America; 9 Université Grenoble Alpes, Saint-Martin-d’Hères, France; 10 martijnroelandse.dev, Ouderkerk aan de Amstel, Netherlands; 11 Hogeschool van Amsterdam, Amsterdam University of Applied Sciences, Amsterdam, Netherlands; 12 Indiana University School of Public Health, Bloomington, Indiana, United States of America; 13 Department of Neuroscience, UC San Diego, La Jolla, California, United States of America; 14 SciCrunch Inc., San Diego, California, United States of America; 15 CIBB, Center for Innovative Biomedicine and Biotechnology, University of Coimbra, Coimbra, Portugal; 16 CNC-UC, Center for Neuroscience and Cell Biology, University of Coimbra, Coimbra, Portugal; University of Queensland - Saint Lucia Campus: The University of Queensland, AUSTRALIA

## Abstract

The causes of the reproducibility crisis include lack of standardization and transparency in scientific reporting. Checklists such as ARRIVE and CONSORT seek to improve transparency, but they are not always followed by authors and peer review often fails to identify missing items. To address these issues, there are several automated tools that have been designed to check different rigor criteria. We have conducted a broad comparison of 11 automated tools across 9 different rigor criteria from the ScreenIT group. We found some criteria, including detecting open data, where the combination of tools showed a clear winner, a tool which performed much better than other tools. In other cases, including detection of inclusion and exclusion criteria, the combination of tools exceeded the performance of any one tool. We also identified key areas where tool developers should focus their effort to make their tool maximally useful. We conclude with a set of insights and recommendations for stakeholders in the development of rigor and transparency detection tools. The code and data for the study is available at https://github.com/PeterEckmann1/tool-comparison.

## Introduction

The reproducibility crisis remains a central concern [1,2] in scientific fields ranging from psychology [3] to cancer biology [4]. The causes of this crisis include lack of standardization and transparency in scientific reporting [5], which has led to the addition of various checklists and instructions for grantees and authors. Popular checklists such as the CONSORT [6] or ARRIVE [7] guidelines for human and preclinical animal studies, respectively, have been proposed and added to prominent journals’ instructions to authors. Funders such as the NIH [8] have also implemented checklists as part of the grant submission process. Checklists may increase awareness of issues affecting reproducibility, but evidence suggests they do not significantly improve reporting quality [9,10].

While reproducibility consists of many factors, perhaps one of the easiest to address is transparency. Transparent, high quality reporting is achievable even if a study is already completed, but was not conducted in a fully rigorous manner. This includes details of the methods used such as blinding, research materials, and placing data and software into locations that are as open as possible and as closed as necessary [11,12]. Peer review can help to address many of these issues, but human reviewers often fail to point out important missing information such as catalog numbers for key resources, and may not comment when a criterion like blinding is absent when it is not commonly reported in the field [13]. Humans are also less likely to flag some problematic practices like plagiarism, which requires searching through millions of documents, and manipulated figures, which requires extensive time, training and experience. Therefore, detecting these practices is best done through automated analysis. Paper mills produce problematic papers at scale, which can overwhelm the traditional peer review system and is contributing to an unprecedented number of retractions [14]. For these reasons, the use of software tools has been proposed to automatically flag missing criteria critical for transparency [15]. Many publishers already use central platforms like the STM Integrity Hub [16] to check submitted papers for fraudulent or suspicious content using automated tools, which would otherwise be difficult to catch via traditional peer review. Other tools employ image and text mining techniques to search for criteria such as blinding, power calculations, randomization, open sourcing of code and data in manuscripts, and checking figure quality [17–20].

**Fig 1:**
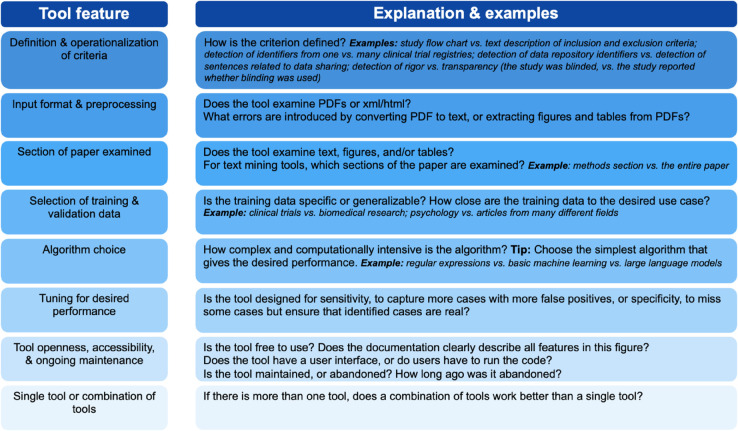
Design features that may affect tool performance. This figure highlights and explains eight features that can affect tool performance,and provides explanations and guiding questions for each feature.

With the expansion of automated tools available to detect rigor criteria, there is an absence of work comparing the efficacy of the tools. Selecting a tool for a given use case can be difficult, as many tools purport to search for the same or similar things. Direct comparisons would help tool developers and users to understand the design and performance differences between tools (examples given in Fig 1). Comparisons would also help users, including publishers, reviewers, and metascientists, to move beyond selecting the tool that performs best in one context, and consider which tool, or combination of tools, fits best with the user’s intended purpose. A comparison would further benefit tool developers to determine which types of tools or which combination of tools are most effective in solving a particular problem, discover areas where existing tools are insufficient, and determine what design decisions affect tool performance.

Most papers that introduce new tools compare performance against similar tools, but the dataset used for these comparisons is not standardized. In the worst case, some tool developers may try multiple datasets and pick the one where their tool has the highest performance, making it even more difficult to assess the tool’s true performance [21]. These practices can lead to “phantom progress,” where each new paper claims to reach state-of-the-art performance, but independent evaluation reveals no differences between old and new methods (e.g. [22]). Thus, a broad, independent comparison of rigor criteria tools is needed.

We seek to address the need by performing a broad, independent comparison across multiple tools and rigor criteria. We built a dataset encompassing a random subset of 1,500 open access manuscripts in PubMed Central, to ensure that our results would be applicable to many biomedical fields. We evaluated 9 rigor criteria, and ran a suite of 11 tools on all papers in our dataset. Human curators assisted in labeling a gold standard dataset for each criterion, primarily focusing on cases where the tools disagreed, which we used to compute performance characteristics for each tool. After applying this standardized approach to many criteria and tools, we use our comparisons to examine progress in tools for detecting rigor criteria and to provide insights for tool developers and users.

## Methods

The tools examined here were created by members of ScreenIT, a community of curators, developers, and other scientists that focus on the problem of scientific rigor and reproducibility [23]. The goal of this community is to alleviate current problems in poor research quality by the independent development of rigor-checking tools. In this study, we analyzed which tools were more and less useful for various criteria. The criteria we chose to address were based on the areas of expertise of the groups within ScreenIT, with each group working on the criteria they were most knowledgeable about. Many of these criteria were selected based on various checklists and guidelines, for example [7,24,25]. An unavoidable side effect is that for many comparisons, the evaluating group had a tool that they developed in the evaluation, but we aimed to reduce bias as much as possible as explained in the following sections.

We attempted to determine which tool performed better, but also considered whether a combination of tools was better suited to address a broader problem in reproducibility. Not all tools are directly comparable because they define the presence or absence of criteria differently; indeed, none of the tools define even seemingly simple things like the presence of code in the same way. For example, some tools assess the availability of open code, while other tools simply detect a statement about code sharing, such as “code is not available for this study.” The sections below contain an overview of the methods used, but see the Supplementary Information for specific methods for each rigor criteria.

### Paper extraction

XML files for 1,500 papers from PubMedCentral’s Non-Commercial Open Access Subset (available at https://ftp.ncbi.nlm.nih.gov/pub/pmc/oa_bulk/oa_noncomm/) with a PMCID starting with PMC008 (approximately March 2021 to April 2022) were selected using a simple random sample. For each paper, both full text (all text contained within the XML’s <body> tag) and the methods section (all text within sections with titles that contain the words “method” or “procedure”) were extracted. Two tools screen PDFs to evaluate figures and tables; therefore, PDFs of all 1,500 papers were also retrieved automatically.

### Test set construction

In general, for each comparison (other than tools that scanned the PDF version of the manuscript), we ran all tools on the extracted full text of the 1,500 papers. However, for some comparisons, the set of 1,500 papers was not used because it would not be very informative. For example, the 1,500 paper corpus does not contain many clinical trial papers, so we would not expect many baseline tables to be present. Instead, we selected a more focused set of trials separate from the main corpus. When we used a different corpus than the main 1,500 set, we will explicitly state that in the section for that comparison.

In cases where all tools agreed on the presence or absence of the criteria of interest, we treated that prediction as the “gold standard” result in our test set and performed no manual labeling. All papers where at least one of the tools disagreed on the presence or absence of a given criteria were examined by a curator assigned to that comparison task. This technique is similar to that of the Cranfield methodology [26,27] from information retrieval, where it is impossible to manually curate every document; therefore, one has to use the tools being studied to extract a smaller set of relevant documents to curate. Human curators were blinded to which tool(s) disagreed. This was done to reduce bias as some curators were developers of the tools being analyzed. In order to speed curation time, we showed the human curator the extracted sentence (e.g. “Code for this paper is available at ... ”) so that curators could quickly identify positive cases. To reduce bias, we randomly selected a tool and displayed its extracted sentence, if it existed. That means that if the selected tool did not find anything, but another tool extracted a sentence, we still showed nothing to the curator.

We sought to estimate the rate of error in our test set where all tools agreed on the classification for a given paper. While it would be infeasible to manually curate every paper where all tools agreed, we randomly sampled 100 papers for each criterion among the papers where all tools agreed and manually labeled them. This was to estimate the rate at which all tools were wrong, so that we could more accurately report performance characteristics of the tools. If we had at least 50 positive (all tools agreed that the criteria was present) and at least 50 negative examples (all tools agreed that the criteria was absent), then the set consisted of 50 positives and 50 negatives. If we had fewer than 50 positive or negative papers, we used more of the other type to reach 100 papers. For all criteria, there were always at least 100 papers where all tools agreed.

### Generic definitions of items

The definition of criteria frequently differed between tools; therefore, curators were instructed to use their best judgment of a criterion’s concept as neutral observers, and not the exact definition that was used by one of the tool makers. This assessment approach was designed to ensure that the criteria were not dependent on any given tool. For example, there are specific definitions of a software tool that were strictly enforced in the training data for SoftCite (Du et al, 2021). These specific definitions were not provided to the curator, who instead used their best guess as to whether the item was or was not a piece of software (more specifics in each analysis subsection). This decision was made to increase the generalizability of the results, but it also introduces a type of bias. Curators tracked their definitions and processes in notes, which are summarized below for each of the analyses.

### Ensemble model

To examine the potential value of combining the tools, a logistic regression ensemble model was trained and tested on the 1,500 paper set for each rigor criteria. The ensemble model was trained to make a classification based on a linear combination of the binary results of the individual tools. Sklearn’s (RRID:SCR_019053) LogisticRegression, trained with cross-entropy loss, was used for this task. Overfitting on the training set was not a concern due to the low expressiveness of a linear model with only four (or less, depending on the number of tools) parameters (plus an intercept). Nonetheless, we assessed overfitting using the method described below in “statistical tests”.

### Statistical tests

Each tool was assessed against the test set using an adjusted accuracy, precision, recall, and F1 score. We used adjusted performance metrics to account for our biased method of assigning ground truth labels. Since we did not manually label papers where no tool predicted a positive, we may be missing positive papers. To estimate the number of positive papers that were missed, we manually labeled a random subset of papers that no tool had predicted as positive. We extrapolated the results from this manual labeling to estimate the true number of positive papers that were missed. This estimate is captured in the “presumed positive rate” (PPR), which is the rate at which papers presumed to be positive were actually positive, and a “presumed negative rate” (PNR), the rate at which papers presumed to be negative were actually negative. We used these rates to compute the following adjusted values:


adjTP=TP*PPR

adjFP=FP*PNR

adjFN=FN*PPR

adjTN=TN*PNR

where TP, FP, FN, and TN are the number of true positive, false positive, false negative, and true negative examples, respectively.

Therefore, the adjusted values will always be more conservative than the unadjusted. The adjusted accuracy, precision (also known as the “positive predictive value”), recall (also known as the “sensitivity”), and F1 score (the harmonic mean of precision and recall) were then calculated as:


$$accuracy=\frac{adjTP+adjTN}{adjTP+adjTN+adjFP+adjFN}$$

$$precision=\frac{adjTP}{adjTP+adjFP}$$

$$recall=\frac{adjTP}{adjTP+adjFN}$$

$$F1=\frac{2}{1/precision+1/recall}$$

For each ensemble model that we trained for a given rigor criterion, we report what we call the “Learned ensemble function” to make the model’s learned decision process transparent. This was done by first inputting all permutations of different binary tool results to the model to generate a truth table. Then, we performed boolean simplification to generate a simplified expression of the function learned by the ensemble.

To assess overfitting of the ensemble models, we trained the ensemble model on random 80% subsets of the training data (80% of the original dataset randomly resampled without replacement), and evaluated which percent of trained models had the same decision function as the model trained on all data. A high percentage indicates that overfitting is unlikely, because the model learns the same function despite the underlying data being different subsets of the larger dataset. Note that while the parameters might be slightly different, the models trained on the subset of data that have the same “Function learned” will have identical test-time behavior.

For comparisons between tools, a two-tailed t-test with variances calculated from sample proportions was used to compare the classification accuracies [28]. Additionally, Gwet’s agreement among each pair of tools, and among each tool to the true results, was calculated using the irrCAC python package (RRID:SCR_023176). Gwet’s statistic measures the agreement between raters whilst adjusting for chance agreement [29]. We did not take into account the presumed positive/negative rate when calculating Gwet’s agreement.

## Results

A summary of the rigor criteria and tools we analyzed, and their important differences, is presented in Table 1. For readability, we summarize the findings from each tool comparison in the below sections. The Supporting information (SI) contains a tabular summary of all comparisons including tool references (S1 Text, Table 1), details for each comparison, as well as performance statistics.

**Table 1:** Overview of individual comparisons. We highlight in overview form each tool comparison that we performed. A dash (“-”) indicates that the tools differed in one of the following ways, and a cross mark (“X”) denotes that the difference made a substantial impact on the final performance. Differences include how the definitions of terms are operationalized into a tool (e.g. does the tool recognize power calculations or other means to check for group size), document input format and requirements for preprocessing (e.g. extraction of text or images from PDF documents can be challenging and introduce systematic errors), section of the paper examined (e.g. related to sensitivity vs specificity as some information may be missed by a tool that does not run on the section of text where the mention appears), selection of training and validation data such as the field (e.g. clinical studies vs psychology), algorithm choice (e.g. regular expressions vs large language models), the openness & accessibility of the tool (e.g. does the tool have a version with a user interface, is the tool maintained, is the tool free and open code), and the desired performance (e.g. the tool may be tuned for sensitivity to capture more cases with more false positives). See Fig 1 for more details on the main differences between tools.

Criterion	Tools included	Operationalization of criteria	Input format & preprocessing	Sections of article	Selection of training & validation data	Algorithm choice	Tool openness & accessibility	Sensitivity vs. specificity	Ensemble useful?
Registration	TRNScreener ctregistries SciScore	X		X	-		-	X	N/A
Inclusion & exclusion criteria	pre-rob Barzooka SciScore	-	X	-	-	X	-		Yes
	
Blinding	CONSORT-TM pre-rob SciScore			-	-	X	-		No
Randomization	CONSORT-TM pre-rob SciScore			-	X	-	-		Yes
Power calculations	CONSORT-TM SciScore	-		-	-	-	-		No
Use of software tools	SoftCite SciScore	X		X	X	-	-		No
Open code	ODDPub SciScore	X		X	X	X	X		No
Use of problematic cell lines	PCL Detector SciScore	X		X	X	X	X	X	No
Baseline tables	baseline_tables Baseline	X	X	X	X	X	X		N/A

**Registration** (S1 Text, Tables 3 and 4): In the comparison of registration tools, which look for clinical trial numbers and other protocol registration numbers, the definition of what the tool aimed to find was a major source of difference in performance. SciScore’s definition includes protocols (2 were found in the dataset) while other screening tools look for different sets of clinical trial registries. By far the most prevalent registry found in the 1500 papers was clinicaltrials.gov (ctgov, S1 Text, Table 4) which was responsible for 137 of 169 total items correctly identified. Interestingly, the ctregistries tool, which recognizes the most registries, also accounted for all false positives in this dataset because the various trial registries often share letter number combinations with granting agency numbers, catalogue numbers and medical abbreviations. We did not consider an ensemble model for this comparison because we compared tools at the entity level (mention of a specific trial identifier) and not at the paper level, so it is unclear how to perform ensembling in this case.

**Inclusion and exclusion criteria** (S1 Text, Tables 5, 6, and 7): The comparison of detection of inclusions and exclusions of participants was tested with three tools: pre-rob, SciScore and Barzooka. The first two tools recognize text using an LLM (large language model) and a CRF (conditional random field) while Barzooka recognizes images of flow charts using a CNN (convolutional neural network model). The raw performance of Barzooka was lower than the other two tools, but that was mostly because authors in our set of 1500 papers primarily described inclusions and exclusions using text, not flow charts. However, the performance of the different tools was also highly complementary. When authors described their inclusions using a flow chart, this information was only detected by Barzooka. When inclusions were described in text, this was usually detected by one of the text tools. As a result, the combination of tools was more effective than any individual tool. The ensemble performance accuracy, precision, recall, and the F1 were all in the 0.95 to 0.98 range where the highest performance, F1, of any individual tool was 0.91.

**Blinding and randomization** (S1 Text, Tables 8, 9, 10, 11, 12, and 13): The comparisons for blinding and randomization were both performed using three tools: pre-rob, SciScore and CONSORT-TM [20]. These three tools all processed text using different models with SciScore using CRF, while the other two tools used language models, specifically two versions of BERT [30]. For the comparison of blinding, SciScore performed the best (F1 of 0.89) and the ensemble did not add any performance (F1 of 0.89). Detecting randomization was more difficult for the models, and performance was far lower for all tools (F1’s of 0.40 to 0.76), perhaps because “random” is used to refer to other techniques beyond random assignment such as random effect models. CONSORT-TM did relatively poorly, while pre-rob and SciScore performed better. In this case, the training dataset is likely to explain the difference in performance. CONSORT-TM was trained on text from randomized controlled trials. pre-rob was trained primarily on data from preclinical animal studies. SciScore was trained on a very broad dataset with many different types of studies. The ensemble model also outperformed any individual tool (F1 of 0.76), demonstrating the benefits of combining tools.

**Sample size determination** (S1 Text, Tables 14, 15, and 16): In the analysis to detect how sample size was determined (e.g. power or sample size calculations), we tested SciScore and CONSORT-TM. These tools have similar performance, with an F1 of 0.79 and 0.78 respectively. The main difference seemed to be the tuning of the tool. CONSORT-TM was more likely to produce false positives, while SciScore was more likely to produce false negatives. The ensemble model did not improve performance.

**Software used** (S1 Text, Tables 17, 18, and 19): To find mentions of software in papers, we tested SciScore and SoftCite. SoftCite scored a higher accuracy and F1 score (F1 of 0.87 compared to 0.27), and the ensemble learned to just use the SoftCite results. Here the difference was mainly due to a large number of false negatives in the SciScore data, which is likely to occur when software is mentioned outside of the methods section. Furthermore, SciScore was tuned to find or suggest RRID type entities; therefore this tool uses the RRID list of existing software (https://rrid.site/data/source/nlx_144509-1/search), and does not detect software that isn’t included in this list. The ensemble model did not increase performance over SoftCite alone.

**Open code** (S1 Text, Tables 20, 21, and 22): For detecting the presence of open code statements, we tested SciScore and ODDPub. We found that the simple regex-based tool, ODDPub, outperformed the machine learning tool SciScore. While the tools had significantly different definitions of what constitutes open code, we found that the majority of differences between the tools were due to mistakes from the SciScore machine learning model. The ensemble model did not increase performance over ODDPub alone.

**Contaminated cell lines** (S1 Text Tables 23, 24, and 25): We compared the ability of SciScore and PCLDetector to flag mentions of contaminated cell lines in papers, as defined by the International Cell Line Authentication Committee (ICLAC) or Cellosaurus. We found that SciScore had a high precision but low recall, while PCLDetector had a high recall but low precision. Thus, the difference between the tools can be mostly attributed to how conservatively they were designed. Depending on the use case, users may reasonably select either tool: PCLDetector if they wish to capture as many contaminated cell lines as possible (e.g. as a tool assisting peer review), and SciScore if they want to ensure that most of the detections are true (e.g. when analyzing problematic cell lines across the literature). The ensemble tool did not add performance over PCLDetector alone.

**Baseline table detection** (S1 Text, Table 26 and Fig 1): For extracting the baseline tables in randomized controlled trials, we compared the tools “baseline” and [unnamed]. baseline uses XML as input, while [unnamed] uses PDF. We found that baseline outperformed [unnamed], primarily because it used the XML of the paper as input, which is easily machine-readable. The PDF-based tool was less accurate because it had to rely on imperfect computer vision to detect tables. Thus, tool makers should aim to use an input format which is easily machine readable, as long as it is available for the papers they wish to run their tool on. We did not consider an ensemble model for this comparison because we compared tools at the entity level (individual baseline tables) and not at the paper level, so it is unclear how to perform ensembling in this case.

## Discussion

In this study, we have conducted a broad comparison of 11 automated tools across 9 different rigor criteria. Based on the results of the comparisons, we draw the following conclusions to guide future tool development.

Firstly, while there are overwhelming performance gaps between tools in some comparisons, most comparisons have marginal differences between the tools. In these cases, tool run times, cost, ease of use, and quality/transparency of the output are likely more important factors than the raw performance. Therefore, tool developers should invest effort in tool usability and transparency in addition to pure performance.

Secondly, success when testing a tool on a different dataset than it was trained on is varied. The pre-rob tool was trained on animal studies, and had strong performance in randomization, but poor performance in inclusion/exclusion detection when used on a dataset of general biomedical papers. Therefore, it is important for developers to make sure they train their tool on a dataset very similar to what it will be applied to. Otherwise, they risk substantially worse performance when applied in practice.

During tool development, toolmakers make crucial decisions about how to define the feature of interest. These decisions are major contributors to performance differences between tools, as is illustrated by the comparison of tools to detect protocols. The SciScore definition of “protocols” includes clinical trials but also methods descriptions that one might find in protocols.io, whereas the other tools are only designed to detect clinical trial registrations. Users who are only interested in clinical trial registrations should use TRANscreener or ctregistries, whereas those who are interested in clinical trial registrations and step-by-step protocols for methods should use SciScore.

Thirdly, combining tools improves performance over any individual tool in some cases. Combining the best performing tool with tools that did not perform as well led to improved performance, compared to the best tool alone. Therefore, users should not dismiss worse-performing tools that detect similar things to another tool with better performance. If a tool performs detection in a different way or was trained on a different dataset, it is likely that it will still identify things that the tool with the best performance will miss. We also found that combining results from different tools is particularly valuable when a rigor criteria can be expressed in different modalities, like images and text. In these cases, ensembling tools that search different modalities can be extremely helpful to improve performance. Therefore, tool developers should consider what modalities rigor criteria can be expressed in and develop tools for modalities other than text, including multi-modal tools.

The primary limitation of our study is the construction of our gold standard set. Since we used the output of the tools to determine which papers humans would manually curate, it is likely we failed to correctly classify papers that all tools classified incorrectly. We sought to mitigate this problem by manually classifying a random sample of papers where all tools agreed, and using the resulting data to adjust our performance statistics. Other limitations of our study include a limited set of tools and a limited set of rigor criteria tested.

Bulleted lists of insights from our study are provided below for each of the key stakeholders in the development of automated rigor and transparency tools.

### Insights for toolmakers

As outlined in Fig 1, toolmakers should consider the following when designing tools, as these decisions can substantially impact performance.

- Operationalization of criteria
- Input format and preprocessing
- Sections of paper examined
- Selection of training and validation data
- Algorithm choice
- Tool openness, transparency and accessibility
- Desired performance: Toolmakers can choose to prioritize sensitivity or specificity, or give users the option to adjust the classification threshold

### Insights for new toolmakers using LLMs

The proliferation of LLMs has prompted many people without coding experience, or other experience developing tools, to start creating tools. In addition to the insights shared for tool makers above, we highlight some specific points for those using LLMs to develop screening tools.

- **Simpler approaches often perform better.** LLMs are inherently complicated, computationally intensive, and very expensive to develop and run. The large energy requirements exacerbate environmental impact. Toolmakers should use the simplest approach possible that achieves the desired performance. Lower complexity reduces computational time, energy needs, costs, and the likelihood of errors in the code. Simpler approaches work especially well for things that are consistently reported in standard ways, in specific sections of the manuscript. The fact that you can use an LLM doesn’t mean that you should.
- **Results may not be reproducible.** LLMs are a black box. Tool developers do not know what criteria they are using to classify, and identical prompts may not give the same response, especially when the LLMs have undisclosed version changes.
- **Tool creators need a stable version that they control.** Otherwise, performance will change each time that the LLM is updated. Tool makers may not know when updates have occurred.
- **Validation is essential.** Many tools are released without any data on performance, which means users have no information on exactly what they are designed to detect, how they were trained, how often they make mistakes/hallucinations, and whether the tool is appropriate for their use case. Validations against human curated gold standard data should be performed regularly, and repeated every time that the tool or underlying LLM is adjusted.
- **Tool makers should consider using a private version of the LLM.** Otherwise, the LLM may use data that users enter to further train the LLM. This is a particular concern if toolmakers enter information that is not already publicly available.

### Insights for tool users

- Pay attention to tool inputs and preprocessing steps.
- Validate the tool on your own dataset to determine whether the tool is appropriate for your use case.
- Test ensemble tools. In some cases, one tool is clearly superior. In other cases, a combination of tools performs better than any individual tool.
- Know the limitations of the tool(s) that you use.
- Consider openness, accessibility, and whether the tool is maintained.

### Insights for those receiving results from tools

Those who receive results or reports from tools should pay particular attention to the following details when using and interpreting the reports.

- **Understand the criteria.** Consult documentation in the report to understand how each item is defined, and why the items that are assessed are important. Readers must understand exactly what the tool was designed to detect, and how the criteria were operationalized, to interpret the report. Understanding why the items are important will help users to improve the paper.
- **Some items may not be relevant.** Remember that some items may not apply to every paper. Some tools simply report that the item wasn’t found, without determining whether it was needed. Other tools distinguish between cases where an item is not reported, and cases where the item is not relevant.
- **Expect errors.** When a tool measures many items, it is likely that you will see at least one false positive or false negative in a report. Even among classifiers with very high performance, the likelihood of an error on at least one item is high.
- **Regular users should validate performance.** If you use reports from tools regularly (e.g., editors) or for larger datasets (e.g., metascientists), check some percentage (∼10%) of the reports manually. All tools make mistakes, and regular users should know the types of errors that the tools make and how often these errors occur. This will help you to use the report responsibly.

## Supporting information

S1 Text Contains detailed information about all comparisons. (PDF)
