# Supplementary material for: Use as directed? A comparison of software tools intended to check rigor and transparency of published work
Source: PLoS One. 2026 Feb 13;21(2):e0342225. doi: 10.1371/journal.pone.0342225 (PMC12904390; doi:10.1371/journal.pone.0342225)
Supplement: S1 Text — (PDF) [file pone.0342225.s001.pdf]

Supporting Information: Use as Directed? A Comparison of Software Tools Intended to Check Rigor and Transparency of Published Work

Peter Eckmann[1], Adrian Barnett[2], Alexandra Bannach-Brown[3], Elisa Pilar Bascunan Atria[3], Guillaume Cabanac[6], Louise Delwen Owen Franzen[3], Małgorzata Anna Gazda[9], Kaitlyn Hair[8], James Howison[11], Halil Kilicoglu[7], Cyril Labbe[4], Sarah McCann[3], Vladislav Nachev[3], Martijn Roelandse[10], Maia Salholz-Hillel[3], Robert Schulz[3], Gerben ter Riet[5], Colby Vorland[12], Anita Bandrowski*[13,14], Tracey Weissgerber*[3,15,16]

1 Department of Computer Science and Engineering, UC San Diego, La Jolla, CA, United States
2 School of Public Health and Social Work, Queensland University of Technology, Kelvin Grove, Australia
3 QUEST Center for Responsible Research, Berlin Institute of Health at Charité Universitätsmedizin Berlin, Germany
4 Université Grenoble Alpes, France
5 Hogeschool van Amsterdam, Amsterdam University of Applied Sciences, Amsterdam, Netherlands
6 Université de Toulouse & Institut Universitaire de France, France
7 School of Information Sciences, University of Illinois Urbana-Champaign, IL, United States
8 UCL Social Research Institute, University College London, London
9 Department of Biological Sciences, University of Montréal,1375 Avenue Thérèse-Lavoie-Roux, H3C 3J7 Montréal, Québec, Canada
10 martijnroelandse.dev, Ouderkerk aan de Amstel, Netherlands
11 Information School of the University of Texas at Austin, Austin, TX, United States
12 Indiana University School of Public Health-Bloomington, IN, United States
13 Department of Neuroscience, UC San Diego, La Jolla, CA, United States
14 SciCrunch Inc.
15 CIBB, Center for Innovative Biomedicine and Biotechnology, University of Coimbra, Coimbra, Portugal
16 CNC-UC, Center for Neuroscience and Cell Biology, University of Coimbra, Coimbra, Portugal

* abandrowski@ucsd.edu
* tracey.weissgerber@uc.pt

1 Supporting Information

Summary of all comparisons

Table 1 shows a summary of all comparisons, and Table 2 shows the learned ensembling function for all comparisons.

Table 1. Summary of results for all comparisons. We report the performance for each tool for each rigor criteria we studied (except for Registration Reporting and Software Tool Detection, which did not follow the same methodology as the other comparisons). The "Total papers analyzed" column reports the total number of papers that were classified by the tools, and "Num. all positive/negative" reports how many of those papers were marked as positives/negatives by all the tools.

Criteria	Total papers analyzed	Num. all positive	Num. all negative	Tool	Accuracy	Precision	Recall	F1
Inclusion/exclusion criteria	1500	84	734	pre-rob (RRID:SCR_025493)	0.69	0.94	0.48	0.63
				SciScore (RRID:SCR_016251)	0.91	0.97	0.86	0.91
				Barzooka (RRID:SCR_018508)	0.61	**1.0**	0.31	0.47
				Ensemble	**0.96**	0.98	**0.95**	**0.96**
Blinding	1500	60	1204	CONSORT-TM (RRID:SCR_021051)	0.95	0.86	0.56	0.68
				pre-rob (RRID:SCR_025493)	0.89	0.45	**0.87**	0.59
				SciScore (RRID:SCR_016251)	**0.98**	**0.94**	0.85	**0.89**
				Ensemble	**0.98**	0.93	0.85	**0.89**
Randomization	1500	60	1020	CONSORT-TM (RRID:SCR_021051)	0.87	0.43	0.37	0.4
				pre-rob (RRID:SCR_025493)	0.91	0.59	0.84	0.69
				SciScore (RRID:SCR_016251)	0.88	0.5	**0.94**	0.65
				Ensemble	**0.94**	**0.73**	0.78	**0.76**
Power analysis	1500	104	1264	CONSORT-TM (RRID:SCR_021051)	0.95	0.68	**0.96**	**0.79**
				SciScore (RRID:SCR_016251)	0.96	0.88	0.7	0.78
				Ensemble	**0.97**	**1.0**	0.66	**0.79**
Open code	1500	12	1438	SciScore (RRID:SCR_016251)	0.98	0.55	0.67	0.6
				ODDPub (RRID:SCR_018385)	**1.0**	**0.95**	**0.92**	**0.94**
				Ensemble	**1.0**	**0.95**	**0.92**	**0.94**
Problematic cell lines	1500	4	1452	PCLDetector (Link)	**0.99**	0.76	**0.74**	**0.75**
				SciScore (RRID:SCR_016251)	0.98	**1.0**	0.36	0.53
				Ensemble	**0.99**	0.76	**0.74**	**0.75**
Baseline table detection	100	51	34	baseline (RRID:SCR_025128)	**0.80**	**0.88**	**0.77**	**0.82**
				[unnamed] [1]	0.67	0.75	0.68	0.71

Table 2. Learned ensemble functions for all comparisons. The "Learned ensembling function" column shows a simplified boolean expression of the function learned by the ensemble model. The "% same with resampled training set" column reports the percentage of trained ensemble models that learn the same boolean function as the one reported when trained on a resampled dataset of 80% the size as the full training set.

Comparison	Learned ensembling function	% same with resampled training set
Inclusion/exclusion criteria	SciScore OR Barzooka	99%
Blinding	(CONSORT-TM AND pre-rob) OR (pre-rob AND SciScore) OR (CONSORT-TM AND SciScore)	95%
Randomization	pre-rob AND SciScore	90%
Power analysis	CONSORT-TM AND SciScore	100%
Software tools	SoftCite	88%
Open code	ODDPub	77%
Problematic cell lines	PCLDetector	82%

Methods and results for individual criteria

Registration reporting

Tools: To detect registration reporting, we used the Trial Registration Number screener (TRNscreener; RRID:SCR_019211; `https://github.com/bgcarlisle/TRNscreener/`

Table 3. List of tools for registration reporting and their reported purpose.

Tool category	Tool name	RRID and commit	Searches for	Search in	Classification method
Clinical trial identification	TRNscreener	RRID:SCR_019211 (commit)	Trial identifier in ClinicalTrials.gov and ISRCTN	Full text	Regex
	ctregistries	RRID:SCR_024412 (commit)	Trial identifier in any WHO ICTRP Primary Registry or Data Provider	Full text	Regex
	"NCT" presence	N/A	Presence of the text "NCT" (to detect trial identifiers in ClinicalTrials.gov, which start with "NCT")	Full text	Regex
Registration identification (including clinical trials in select registries)	SciScore	RRID:SCR_016251, version 2	Trial identifier in ClinicalTrials.gov and EU Clinical Trials Register; Identifier from Protocols.io, Protocol Exchange, STAR Protocols, JoVE, Bio-protocol, MethodsX, Nature Protocols, Spring Protocols, BioTechniques,and PROSPERO	Methods section	Regex

Table 4. Registration identifiers found in papers. True positives indicate the number of each type of registration identifier found by any tool and then manually validated. An empty cell indicates that the tool was not built to detect that type of identifier. Registries: [1]

	True positives	SciScore		ctregistries		TRNscreener		nct	
	n	n	%	n	%	n	%	n	%
TRN (total)	168	55	32.5%	166	98.2%	139	82.2%	136	81.1%
ctgov	136	54	39.4%	134	97.8%	136	99.3%	136	100%
umin	7			7	100%				
drks	5			5	100%				
irct	5			5	100%				
chictr	3			3	100%				
isrctn	3			3	100%	3	100%		
ctri	2			2	100%				
eudract	2	1	50%	2	100%				
actrn	1			1	100%				
jrct	1			1	100%				
kct	1			1	100%				
ntr	1			1	100%				
pactr	1			1	100%				
Protocols (total)	2	2	100%						
FP (total)	29			29	100%				
funding_id	13			13	100%				
drug_id	6			6	100%				
datapoint	5			5	100%				
catalog_id	3			3	100%				
medical_acronym	1			1	100%				
medical_device	1			1	100%				

`commit/9d02549a8d2ede3995013c8347aa33e0c9220427`), ctregistries (RRID:SCR_024412; `https://github.com/maia-sh/ctregistries/commit/06c169cfa241ef8feda9e8b78f57b3012c85afd8`), SciScore v2 (RRID:SCR_016251), and PE's naive tool (Table 3). This tool checked for the presence of "NCT", the prefix for ClinicalTrials.gov identifiers, anywhere in the paper. These tools were applied to the full text extracted from each of the 1500 PMC papers in the test set. All tools searched for trial registration numbers from one or more trial registries, yet the tools differed with regard to which registries were considered, ranging from ClinicalTrials.gov only for the naive "NCT" tool, to all registries in the World Health Organization (WHO) International Clinical Trials Registry Platform (ICTRP) Primary Registries and Data

Providers for ctregistries. SciScore considers trial registries akin to protocols, thus additionally finds identifiers for protocol repositories such as protocols.io, which is a case where the definition of “registry” is likely to be a large portion of the difference in performance. In cases where tools extracted multiple identifiers per paper, all identifiers were captured, duplicates were removed, and the remaining unique identifiers were sorted alphabetically to make for easy comparison if tools extracted identifiers in different orders. Duplicate identifiers found across different papers were preserved. This resulted in a dataset of unique identifiers per paper.

Manual checks: One co-author (EB) coded papers, and questions were discussed with two other team members (DLF and MSH). All papers where any tool detected an identifier were manually checked. For each identifier found by any tool, the coder located the unique identifier in the full-text paper; the paper was not checked for further identifiers, and identifiers were not validated (i.e., the registry was not checked for a corresponding registration). Each identifier was classified as a trial registration number, protocol identifier, or false positive. For trial registration numbers and protocol identifiers, the registry or repository of the identifier was captured. For false positives, we noted what the number represented (e.g. funding IDs, medical acronyms, drug IDs). The coder annotated all of the identifier’s locations in the paper, including: abstract, methods or other locations (e.g., introduction, acknowledgments, discussion, etc.). Finally, the coder classified the paper as a research article or another type of paper.

Additionally, a random sample of 50 papers for which no tools found any identifier were manually reviewed in their entirety for the presence of an identifier. The coder scanned the full text of each paper in search of a unique identifier. Any possible identifiers were noted and were reviewed by two additional team members to determine whether any tools should have found the identifier (i.e., false negative).

We chose not to conduct a performance analysis for this comparison, because differences were relatively minor between tools. Instead, we conducted a descriptive analysis of the differences between the tools, which we believe better captures the relevant differences between the tools.

Descriptive analysis of identifiers: After running all tools across the 1,500 paper test set, a total of 199 hits for unique registration identifiers unique per paper were found by one or more tools in 117 papers. TRNscreener found 139 hits across 72 (4.8%) papers, ctregistries 195 across 113 (7.5%), SciScore 57 across 43 (2.9%), and “NCT” was present 136 times across 67 papers (4.5%). Results are summarized in Table 4.

In our manual checks of the 199 identifiers, we found that 168 were trial registration numbers (TRN), 2 were protocol identifiers, and 29 were false positives (e.g., grant number). The majority of TRN were from ClinicalTrials.gov (136), followed by the Japanese University Hospital Medical Information Network Registry (7), the German Clinical Trials Register (5), and the Iranian Registry of Clinical Trials (5). Additionally, there were nine additional trial registries with fewer than 5 hits per registry.

Taken together, each tool found different types of IDs. SciScore found mainly TRN from ClinicalTrials.gov (n = 54) and the EU Clinical Trials Register (n = 1). It was also the only tool that found two protocol IDs (n = 2). Note that unlike the other tools, SciScore only searched for identifiers in the Methods sections of papers, so it would miss all other identifiers not in the Methods. Nct found only TRNs from ClinicalTrials.gov (n = 136). TRNscreener found TRNs from ClinicalTrials.gov (n = 136) and the ISRCTN Registry (n = 3). Finally, ctregistries found TRN from several different registries (n =

[1] ctgov - ClinicalTrials.gov Registry, umin - University Hospital Medical Information Network Registry, drks - German Clinical Trials Register, irct - Iranian Registry of Clinical Trials, chictr - Chinese Clinical Trial Registry, isrctn - ISRCTN Registry, ctri - Clinical Trials Registry-India, eudract - EU Clinical Trials Register, acTRN - Australian New Zealand Clinical Trial Registry, jrct - Japan Registry of Clinical Trials, kct - Korean Clinical Trial Registry, ntr - Dutch Trial Register, pactr - Pan African Clinical Trials Registry.

166), where the majority were identifiers from ClinicalTrials.gov (n = 134). This tool also found the 29 cases of false positives, where the majority were funding IDs (n = 13).

Finally, it is important to note that there were several papers in the main set that used the registration IDs to cite previous works, mainly in the introduction/background section of the paper. This is relevant as finding an ID by the tools on the papers does not automatically translate to the fact that a study mentioned its own registration id as "good practice". For example, in this analysis, 71 out of the 136 ClinicalTrials.gov trial registration numbers were used as reference to other studies.

Additionally, a manual check was conducted on a set of 50 PMC papers where no registration identifier was found by any of the tools. In this set there was no registration id from the registries mentioned above. However, there were three cases where a different type of registration was discovered: a prospero registry ID (CRD42021225699), a number ID from a registry website (researchregistry7399), and a number ID from the Institutional Animal Care and Use Committee (2014092403).

Inclusion/exclusion criteria

Table 5. List of tools for inclusion/exclusion criteria detection.

Tool category	Tool name	Searches for	Method of classification
Inclusion/exclusion criteria detection	pre-rob	Sentence(s) including inclusion/exclusion criteria	BERT encoding + linear classifier
	SciScore	Sentence(s) including inclusion/exclusion criteria	CRFs
	Barzooka	Images of flow diagrams	CNN

Table 6. Estimated tool performance statistics for inclusion and exclusion criteria detection. Statistics are estimated based on the gold standard test set of 1,500 papers (see Methods for details). The Ensemble tool uses a combination of the results from all tools to make a classification.

Tool	Accuracy	Precision	Recall	F1
pre-rob	0.69	0.94	0.48	0.63
SciScore	0.91	0.97	0.86	0.91
Barzooka	0.61	**1.0**	0.31	0.47
Ensemble	**0.96**	0.98	**0.95**	**0.96**

Tools: The output of three tools used to detect inclusion and exclusion criteria was examined: pre-rob (RRID:SCR_025493, `https://github.com/qianyingw/pre-rob/commit/c0f3fe56c2706e89a7b8bcf151b29dce8ddbbd45`), Barzooka (RRID:SCR_018508, `https://github.com/quest-bih/barzooka/commit/8ab662db3f2cc4b691f17bf508956952260d58c6`) and SciScore (RRID:SCR_016251) (Table 5). Barzooka was run on PDF documents because it detects images, specifically flow diagrams that define the experimental exclusions, whilst the other tools used the full text extracted from XMLs.

Curation: We performed manual validation of 679 (+100 controls) articles. This subset was comprised of all 679 articles on which any of the tools disagreed and 100 randomly selected control articles where the tools agreed. The human curator for text

Table 7. Gwet's agreement among each tool and the gold standard ("true") for inclusion and exclusion criteria.

	pre-rob	SciScore	Barzooka	Ensemble	True
pre-rob	1.0	0.51	0.60	0.44	0.49
SciScore		1.0	0.38	0.91	0.84
Barzooka			1.0	0.44	0.42
Ensemble				1.0	0.92
True					1.0

(AEB) broke up the sentences into three possibilities: Yes, No and 'it is complicated'. The sentences recognized by the tool were rated on the criteria that they did or did not represent inclusion or exclusion criteria. Sentences that started with "The exclusion criteria were ..." or similarly clear statements were not carefully checked but simply accepted as true. Sentences that clearly included study patients or participants, and words that indicated inclusion or exclusion were also marked as "yes", while sentences that were clearly about excluding or including some experimental variable that was not about the subject population were marked with "no", for example, "To alleviate artifacts caused by head motion, subjects with mean frame-to-frame displacement > 0.1 mm or maximum frame-to-frame displacement > 0.15 mm were excluded." In the third category where it was not clear to the curator for 26 sentences whether the exclusion was associated with the subject population or not, for example, "A variable to be analyzed was excluded from the covariates." In these cases, the curator reviewed the sentence a second time to determine whether the sentence could be clarified. This removed all but 12 borderline statements. For data not directly associated with a particular sentence, the manuscript was examined by first searching for "inclusion" or "exclusion" and then looking for flow diagrams. The human curator for flow charts (TLW) reviewed all flow charts detected by the tool to determine whether they were flow charts depicting the number of included and excluded observations at each stage of the experiment, and explaining reasons for exclusion.

Positive or ambiguous text statements were pasted into the same document as evidence of the presence of the criterion for documentation. Flow charts were not pasted as they were too big.

Results: Tables 6 and 7 report the performance of each tool as well as an ensemble model combining all the tools. While SciScore had a significantly higher accuracy and F1 over pre-rob and Barzooka, the ensemble model was able to further improve the classification performance. Interestingly, the ensemble learned the function (SciScore OR Barzooka). This shows that the performance boost of the ensemble over SciScore is because the ensemble is able to take both image and text modalities into account. These results indicate the importance of ensembling multiple tools together in cases where criteria may appear in multiple modalities, such as text and image.

Blinding

Tools: To detect blinding we used three tools, CONSORT-TM (RRID:SCR_021051, `https://github.com/kilicogluh/CONSORT-TM/commit/b40a4f9e354084b693578d110c51468138608f7a`), SciScore (RRID:SCR_016251), and pre-rob (RRID:SCR_025493, `https://github.com/qianyingw/pre-rob/commit/c0f3fe56c2706e89a7b8bcf151b29dce8ddbbd45`) (Table 8). All three tools are text based.

Curation: We performed manual validation of 291 (+100 controls) articles. This

Table 8. List of tools for blinding detection.

Tool category	Tool name	Searches for	Method of classification
Blinding	pre-rob	Sentence(s) including blinding	BERT encoding + linear classifier
	SciScore	Sentence(s) including blinding	CRFs
	CONSORT-TM	Sentence(s) including blinding	BioBERT-based classifier

Table 9. Estimated tool performance statistics for blinding detection. Statistics are estimated based on the gold standard test set of 1,500 papers (see Methods for details). Performance values are adjusted based on the estimated percentage of papers that are misclassified despite all tools agreeing. The Ensemble tool uses a combination of the results from all tools to make a classification.

Tool	Accuracy	Precision	Recall	F1
CONSORT-TM	0.95	0.86	0.56	0.68
pre-rob	0.89	0.45	**0.87**	0.59
SciScore	**0.98**	**0.94**	0.85	**0.89**
Ensemble	**0.98**	0.93	0.85	**0.89**

Table 10. Gwet's agreement among each tool and the gold standard ("true") for blinding.

	CONSORT-TM	pre-rob	SciScore	Ensemble	True
CONSORT-TM	1.0	0.81	0.94	0.95	0.94
pre-rob		1.0	0.85	0.86	0.86
SciScore			1.0	0.99	0.98
Ensemble				1.0	0.98
True					1.0

subset was comprised of all 291 articles on which any of the tools disagreed and 100 randomly selected control articles where the tools agreed. The human curator (KH) determined whether there was any form of blinding present in the study and assigned a "Yes" or "No" category. As blinding is possible at many different stages, the curator also assigned the papers with some form of blinding into four categories to indicate which parts of the study were blinded (blinded conduct - Yes / No / NA, blinded assessment - Yes / No / NA, blinded scoring between investigators - Yes / No / NA), and whether the method of blinding was described (Yes / No). The NA categories were assigned when the form of blinding addressed by the category was not feasible due to the study type. For example, "blinded conduct" is not possible in a retrospective observational study.

Results: CONSORT-TM, pre-rob, and SciScore were run on the 1500 paper set to detect blinding (Tables 9 and 10). SciScore showed the best overall performance. This may be due to the fact that the other LLM-based tools were trained on a more limited dataset and failed to generalize to the broader literature. The ensemble model achieved a similar performance to SciScore, although notably it learned a complex function of the different tools. In this case, it seems that the extra complexity of the ensemble model is not beneficial to prediction. This suggests that ensembling is not always the best

approach if the learned function is complex and does not improve prediction by much.

Randomization

Table 11. List of tools for randomization detection.

Tool category	Tool name	Searches for	Method of classification
Randomization	pre-rob	Sentence(s) including randomization	BERT encoding + linear classifier
	SciScore	Sentence(s) including randomization	CRFs
	CONSORT-TM	Sentence(s) including randomization	BioBERT-based classifier

Table 12. Estimated tool performance statistics for randomization detection. Statistics are estimated based on the gold standard test set of 1,500 papers (see Methods for details). Performance values are adjusted based on the estimated percentage of papers that are misclassified despite all tools agreeing. The Ensemble tool uses a combination of the results from all tools to make a classification.

Tool	Accuracy	Precision	Recall	F1
CONSORT-TM	0.87	0.43	0.37	0.40
pre-rob	0.91	0.59	0.84	0.69
SciScore	0.88	0.5	**0.94**	0.65
Ensemble	**0.94**	**0.73**	0.78	**0.76**

Table 13. Gwet's agreement among each tool and the gold standard ("true") for randomization.

	CONSORT-TM	pre-rob	SciScore	Ensemble	True
CONSORT-TM	1.0	0.74	0.68	0.79	0.82
pre-rob		1.0	0.8	0.94	0.88
SciScore			1.0	0.86	0.83
Ensemble				1.0	0.92
True					1.0

Tools: We used the CONSORT-TM (RRID:SCR_021051, `https://github.com/kilicogluh/CONSORT-TM/commit/b40a4f9e354084b693578d110c51468138608f7a`), pre-rob (RRID:SCR_025493, `https://github.com/qianyingw/pre-rob/commit/c0f3fe56c2706e89a7b8bcf151b29dce8ddbbd45`), and SciScore (RRID:SCR_016251) (Table 11) to determine if randomization was present. All of these tools are text based and look for sentences that describe randomization of subjects into groups.

Curation: We performed manual validation of 461 (+100 control) articles. This subset was comprised of all 461 articles on which any of the tools disagreed and 100 randomly selected control articles where the tools agreed. The human curators (MAG, AB) assigned sentences into "Yes" and "No" categories. The data were analyzed based on either the sentence extracted by one of the tools, or by manual search using "rando". The data were classified as "Yes" when randomization was used to split a study cohort

into groups, and "No" when there was no randomization or randomization was used for some other purpose (e.g. imputing missing data or cross-validation).

Results: CONSORT-TM, which was trained on randomized controlled trial study data, did relatively poorly. pre-rob, which was trained primarily on animal data, and SciScore, which was trained on a very broad dataset, showed a comparably stronger performances (Tables 12 and 13). The ensemble model had a higher accuracy and F1 than any individual tool. In this case, the training dataset is likely to be the key difference for performance on this broad dataset of randomly selected manuscripts. In this case, the ensemble does much better than any individual tool, showing the benefits of the ensemble approach.

Power calculations

Table 14. List of tools for power calculation detection.

Tool category	Tool name	Searches for	Method of classification
	SciScore	Sentence(s) including power calculations	CRFs
	CONSORT-TM	Sentence(s) including power calculations	BioBERT-based classifier

Table 15. Estimated tool performance statistics for power analysis detection. Statistics are estimated based on the gold standard test set of 1,500 papers (see Methods for details). Performance values are adjusted based on the estimated percentage of papers that are misclassified despite all tools agreeing. The Ensemble tool uses a combination of the results from all tools to make a classification.

Tool	Accuracy	Precision	Recall	F1
CONSORT-TM	0.95	0.68	**0.96**	**0.79**
SciScore	0.96	0.88	0.7	0.78
Ensemble	**0.97**	**1.0**	0.66	**0.79**

Table 16. Gwet's agreement among each tool and the gold standard ("true") for power analysis.

	CONSORT-TM	SciScore	Ensemble	True
CONSORT-TM	1.0	0.89	0.91	0.94
SciScore		1.0	0.98	0.95
Ensemble			1.0	0.96
True				1.0

Tools: To detect power calculations we used two tools, CONSORT-TM (RRID:SCR_021051, `https://github.com/kilicogluh/CONSORT-TM/commit/b40a4f9e354084b693578d110c51468138608f7a`) and SciScore (RRID:SCR_016251) (Table 14).

Curation: We performed manual validation of 132 (+100 controls) articles. This subset was comprised of all 132 articles on which any of the tools disagreed and 100

randomly selected control articles where the tools agreed. Additionally, there were 100 control articles where the tools agreed, including articles where at least one tool detected a statement on power calculations and articles where neither tool identified a statement on power calculation. Each article was assessed by a human curator (RS) to assess the presence or absence of a statement regarding a priori power calculation. Any sentence that indicated that an a priori power- or sample size calculation was performed (e.g., "a priori power calculation present") was rated as "Yes". The criteria did not assess whether the authors reported a minimum set of information that is required for an informative a priori power calculation (e.g. expected effect size, effect size justification, outcome of interest, Type I and II error). Statements on post hoc power calculation and other forms of power calculation that were not done a priori with the aim to estimate a required sample size were excluded, as were statements that no power or sample size calculation were conducted ("a priori power calculation not present").

Results: Both tools reach comparable accuracies, while SciScore has a higher precision and CONSORT-TM has a higher recall (Tables 15 and 16). The ensemble achieves the best accuracy and precision, although it is comparable to the other tools. In this case, the ensemble's increase in accuracy is relatively modest, showing that ensemble approaches do not always lead to large increases in accuracy.

Software tools

Table 17. List of tools for software tool detection.

Tool category	Tool name	Searches for	Method of classification
Software tools	SoftCite	Sentence(s) including software tools	BERT encoding + linear classifier
	SciScore	Sentence(s) including software tools	CRFs

Table 18. Estimated tool performance statistics for software tool detection. Statistics are estimated based on the gold standard test set of 1,500 papers (see Methods for details). Performance values are adjusted based on the estimated percentage of papers that are misclassified despite all tools agreeing. The Ensemble tool uses a combination of the results from all tools to make a classification.

Tool	Accuracy	Precision	Recall	F1
SciScore	0.25	0.58	0.17	0.27
SoftCite	**0.80**	**0.86**	**0.88**	**0.87**
Ensemble	**0.80**	**0.86**	**0.88**	**0.87**

Tools: SoftCite (RRID:SCR_024411, `https://github.com/softcite/software-mentions/commit/05bc633d607831f898902e8bbcbcad120264f5bd`) and SciScore (RRID:SCR_016251) were run to detect whether software was found in the paper (Table 17). For the analysis of SciScore's software tool detector, we extracted all existing or suggested Research Resource IDentifiers (RRIDs) from SciScore's "Key Resources" table and checked for the presence of any RRIDs that corresponded to software or databases as reported in the SciCrunch Registry, a cooperative project aiming to catalog scientific resources (Ozyurt et al. 2016). Software resources were queried for using the "additional resource type" field for any "software", the file we

Table 19. Gwet's agreement among each tool and the gold standard ("true") for software tool detection.

	SciScore	SoftCite	Ensemble	True
SciScore	1.0	-0.91	-0.91	-0.50
SoftCite		1.0	1.0	0.70
Ensemble			1.0	0.70
True				1.0

generated on 28-June-2022 included 7,230 software tools. A live list is accessible via the scientific tools registry at `https://rrid.site/data/source/nlx_144509-1/search?q=%2A&l=&sort=desc&column=Mentions%20Count&sort=desc&filter[]=Resource%20Type:software`. The most commonly used software tools mentioned in papers are statistical tools, such as SPSS or R, and image manipulation tools, such as Photoshop or ImageJ. Both open and commercial tools are included. The list of databases was obtained from the same registry, querying the additional resource types field for "database" on 28-June-2022. This list included 2,970 databases.

Curation: We performed manual curation of 1498 mentions. The human curator (AN) systematically classified entities to be "software" or "not software". Three columns were updated during the curation. The first column was the 'Decision Column', where each entity was rated as "yes", "no", or "it's complicated". The other two columns contained notes regarding the entity, whether it had an RRID or not, and some notes about the entity for "it's complicated" cases.

In the first pass of all the rows, the entity was given a "yes" if it was a software entity. There are two steps to come to that conclusion. The initial classification involves checking if the entity was a URL and, if so, determining its relevance to software based on redirection. For non-URL entities, a detailed classification process considers the presence of code, GitHub repositories, versions, packages and the ability to download the code. If it had code associated with it and had applications of software techniques, it was considered a software entity and marked as yes.

The curator followed an exclusion protocol to identify entities that are not software. The protocol provides specific exclusions, such as entities related solely to databases, search functionality web pages, core facilities, organizational names, and hardware components without associated code or software control. Additionally, entries that are just company names and do not have direct software/code associated with them are not considered software. All these entities were marked no in the decision column. A few entries which were ambiguous were marked as complicated. These entities were revisited as a group in the second pass of all the rows to reach a decision.

Results: SoftCite has a significantly higher accuracy, precision, recall, and F1 compared to SciScore (Tables 18 and 19). A significant contributor to this difference is likely that SciScore only considers the methods section, while SoftCite looks at the entire text of the paper.

Open code

Tools: ODDPub (RRID:SCR_018385, `https://github.com/quest-bih/oddpub/commit/617511915c08e73c47b4c9d829b90f9687a12aa0`) and SciScore (RRID:SCR_016251) were run for each paper's full text and methods section (Table 20). Results were stored in a SQLite database. ODDPub explicitly classifies papers as having open code. SciScore results were based on the presence of "Code Information" in

Table 20. List of tools for open code detection.

Tool category	Tool name	Searches for	Method of classification
Open code detection	SciScore	SciScore Code availability statement OR code identifier	CRF OR regex
	ODDPub	Code identifier AND open access AND result replication possible AND machine readable AND not reused	Regex

Table 21. Estimated tool performance statistics for open code detection. Statistics are estimated based on the gold standard test set of 1,500 papers (see Methods for details). Performance values are adjusted based on the estimated percentage of papers that are misclassified despite all tools agreeing. The Ensemble tool uses a combination of the results from all tools to make a classification.

Tool	Accuracy	Precision	Recall	F1
SciScore	0.98	0.55	0.67	0.6
ODDPub	**1.0**	**0.95**	**0.92**	**0.94**
Ensemble	**1.0**	**0.95**	**0.92**	**0.94**

Table 22. Gwet's agreement among each tool and the gold standard ("true") for open code.

	SciScore	ODDPub	Ensemble	True
SciScore	1.0	0.97	0.97	0.98
ODDPub		1.0	1.0	1.0
Ensemble			1.0	1.0
True				1.0

the rigor table.

Curation: We performed manual validation of 98 (+100 control) articles. This subset was comprised of all 98 articles on which any of the tools disagreed and 100 randomly selected control articles where the tools agreed. The human curator (PE) broke papers into "yes" or "no" categories for the presence of open code. Each paper was first searched for the presence of the words "github", "code", and "available at". Then, each sentence that contained those words was analyzed. We only counted papers as "open code" if they mentioned code that was written, at least in part, by the authors and deposited on a software repository. Cases where authors merely referenced another open source code repository were not counted. If the authors mentioned a repository that contained mostly data, PE viewed the repository and checked for the presence of any associated code to make the final decision. After analyzing all the sentences that contained the above keywords, the curator also examined the beginning and end of the paper's main text to find any statements about open code, as many journals place this information in data and code availability statements. If the paper contained a statement about open code, the curator only counted the statement if it referenced a code repository. Statements like "No original code was generated for this study" were not counted as open code statements.

After running all tools, all cases where one or more tools disagreed over the presence of open code were analyzed manually to make a final determination about the presence of open code. Open code was declared present when 1) the authors explicitly stated where code could be found in the body text (not in a supplementary file), 2) the data/code was hosted on a open repository ("data is available upon request" was not counted), and 3) the data/code was the work of the authors and was released along with the paper (mentioning a third-party repository was not counted).

Results: Performance characteristics of all tools for open code detection on full texts are presented in Tables 21 and 22. The ensemble method learned to just use the output from ODDPub for its prediction, meaning SciScore provided no additional information beyond what ODDPub already provides. While ODDPub and SciScore had generally very similar predictions (Table 22), we selected 10 random papers where the tools reported different results (among the 98 that were curated) and analyzed differences to determine why the tools differed. For 9 (90%) of these papers, SciScore reported open code while ODDPub did not. Among these, 2 (22%) were due to differences in what the tools were designed to do (i.e. both tools were "correct" according to their own reported purposes), 2 (22%) were due to misclassifications by ODDPub (i.e. the sentence was not picked up by ODDPub's regexes), and 5 (56%) were due to misclassifications by SciScore (i.e. SciScore's conditional random field [CRF] model misclassified a sentence). For the one paper (10%) where SciScore did not report open code while ODDPub did, SciScore incorrectly did not find an open code statement. Overall, 80% of the difference between tools is attributable to the ability of the respective classification tools, with 75% of this difference arising from incorrect classifications by a machine learning model. This indicates that model choice and data curation, and even the decision to use a machine learning approach, are more important than the exact definitions of open code used by the tool developers, even if such definitions are very different.

Contaminated cell lines

Table 23. List of tools for contaminated cell line detection.

Tool category	Tool name	Searches for	Method of classification
Contaminated cell lines	PCLDetector	Contaminated cell lines	Embedding + CNN
	SciScore	SciScore Detects cell line sentences, and RRIDs, if an RRID is present and the cell line contains a warning, the warning is displayed	CRFs

Table 24. Estimated tool performance statistics for problematic cell line detection. Statistics are estimated based on the gold standard test set of 1,500 papers (see Methods for details). Performance values are adjusted based on the estimated percentage of papers that are misclassified despite all tools agreeing. The Ensemble tool uses a combination of the results from all tools to make a classification.

Tool	Accuracy	Precision	Recall	F1
PCLDetector	**0.99**	0.76	**0.74**	**0.75**
SciScore	0.98	**1.0**	0.36	0.53
Ensemble	**0.99**	0.76	**0.74**	**0.75**

Table 25. Gwet's agreement among each tool and the gold standard ("true") for problematic cell lines.

	PCLDetector	SciScore	Ensemble	True
PCLDetector	1.0	0.97	1.0	0.99
SciScore (+ manual curation)		1.0	0.97	0.98
Ensemble			1.0	0.99
True				1.0

Tools: SciScore (RRID:SCR_016251) and PCLDetector (`https://gricad-gitlab.univ-grenoble-alpes.fr/nanobubbles/pcl_detector/-/tree/8a9b134c38008e1a70aea026be6a69f9dd89af02`) were used to detect mentions of problematic cell lines (Table 23). SciScore aims at detecting any kind of cell lines, whereas PCLDetector is only designed to detect problematic cell lines tabulated by the International Cell Line Authentication Committee (ICLAC) or Cellosaurus (filtering for comments of type 'problematic cell line'). PCLDetector is based on the ICLAC Register of Misidentified Cell Lines (Version 10) and a dump of Cellosaurus (Version 34.0). The article text is processed by the scispaCy library loaded with the 'en_ner_jnlpba_md' named entity recognition model. As a result, entities of type DNA, CELL_TYPE, CELL_LINE, RNA, and PROTEIN are tagged. Then, the tool searches for the detected cell lines and cell types in the list of problematic cell lines (ICLAC or Cellosaurus). Both tools were run for each paper's methods section.

Curation: Manual curation of each extracted cell line was done by human curators (CL, GC). The curator assumed that if a cell line was listed as problematic in ICLAC, Cellosaurus, or SciCrunch, it was indeed problematic.

Some cell lines are known to be cross-contaminated or otherwise misidentified but are still wrongly used and described in many scientific publications [2, 3]. The use of RRIDs to identify cell lines has shown to be effective in reducing such detrimental usage [4]. Nevertheless most cell line mentions do not feature a RRID. This is why automatically screening scientific texts to specifically detect problematic cell lines is a way to increase the quality of published paper.

Results: After running all tools on the 1500 paper set, PCLDetector found 48 papers (3%) with potentially problematic cell lines. However, SciScore only marked one paper as having problematic cell lines. While SciScore correctly identified two cell lines in this paper as problematic, PCLDetector only found one of these two cell lines. However, as this single match was not enough for a meaningful comparison, we decided to instead manually examine (CL, GC) all cell lines extracted by SciScore (not just those explicitly marked as problematic), and manually look for problems in the suggested cell line entry in SciCrunch. This is a natural step for users who would use the SciScore report, but the lookup to SciCrunch is not integrated into SciScore due to false positive concerns. Using this procedure, SciScore with the manual curation step resulted in 14 papers (1%) marked as having potentially problematic cell lines.

Tables 24 and 25 show the performance characteristics of each tool (including SciScore with the manual curation step) in their ability to detect problematic cell lines. As shown, PCLDetector is able to identify many more problematic cell lines than the modified SciScore, although it has a higher rate of false positives. As PCLDetector is specifically designed to search for problematic cell lines, it is unsurprising that it has a higher recall than a tool which is designed to be more conservative. We also see that the ensemble tool does not improve performance over PCLDetector by itself.

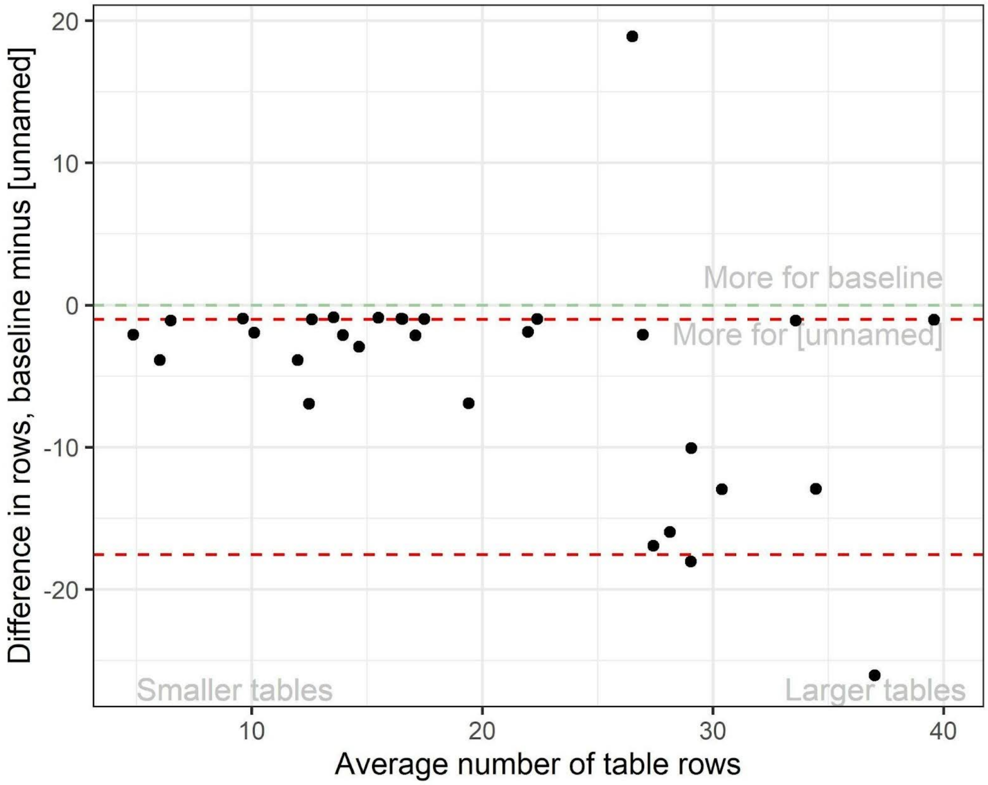

Fig 1. Bland–Altman plot of the number of rows in the baseline table extracted by the [unnamed] and baseline tools. The dotted red lines are the 95% limits of agreement. The dotted green line at zero shows perfect agreement in table row numbers.

Baseline table detection 332

Table 26. Estimated tool performance statistics for baseline table detection.

Tool	Accuracy	Precision	Recall	F1
baseline	**0.98**	**0.98**	**0.98**	**0.98**
[unnamed]	0.89	0.96	0.85	0.90

Tools: We compared two tools that aim to extract the baseline table from randomized controlled trials, baseline (RRID:SCR_025128) [5] and [unnamed] [1]. baseline searches the XML for key phrases that indicate a table comparing randomized groups at baseline using regex. [unnamed] searches the PDF for key phrases that indicate a table comparing groups at baseline using computer vision to extract tables and regex matching on the text. The CONSORT guidelines (`https://www.equator-network.org/reporting-guidelines/consort/`) for randomized trials recommend that papers reporting the results of a trial include a table displaying the characteristics of participants at baseline. The two tools were applied to a random sample of 100 papers that were sampled from [6].

Curation: We did not use the 1500 paper test set because it contained many

studies that were not trials and it was more useful to compare the tools' ability to extract baseline tables in a set of papers that were likely to include many trials. Each paper was manually classified (AB, CJV) as including a baseline table or not, with group discussion used to resolve papers where it was unclear.

Results: The 'baseline' tool did better on accuracy, precision and recall (Table 26), however we should be cautious with these results as the sample size of papers is much smaller than the previous analyses, and different publication formats were used (i.e., XML vs. PDF). The main reasons that the XML-based algorithm excluded baseline tables were because: the table included follow-up comparisons, meaning the z-value distribution would no longer be expected to be centered on zero; the sample sizes could not be extracted; or because the tool did not classify the paper as a randomized trial.

The baseline tool extracted tables using XML and the [unnamed] tool using the PDF. In addition to comparing whether the tools correctly extracted a baseline table or not, we also compared the size of the table extracted using a Bland–Altman plot of the number of rows. The plot shows that using the PDF meant the extracted tables were generally larger with more rows captured, and this was particularly noticeable for larger tables (Figure 1). This likely occurred because some baseline tables were formatted as multiple tables in the XML although they appeared as one table when using the PDF. Smaller differences will have occurred because the XML algorithm only selects one row when there are two perfectly negatively correlated rows, e.g., summary statistics per randomized group on the number of men and women. While the baseline tool performs best, it should be noted that it only works on papers with XMLs available, which is not the case for many non-PubMed Central papers.

Protocols

Ensembling method (applicable to each comparison)

1. For each of the 1500 papers, store the binary output from each tool in an array, as well as the gold standard result from the test set
   (a) The array should have the shape (1500, number of tools)
2. Train a support vector machine (SVM) classifier using sklearn on the full set of papers using the default hyperparameters
3. Use the model to predict the results for the same 1500 paper set, which makes up the output for the "Ensemble" tool
4. Get learned formula
   (a) Generate a truth table for all possible combinations of binary tool outputs, then use the SVM to predict the outcome
   (b) Perform Boolean simplification on the truth table to generate a simplified equation
5. Get % same with subsets
   (a) Take input array and randomly sample half of the entries without replacement, and sample same indices in the output array
   (b) Train SVM using this smaller subset, and perform (4) for each subset and count what percent of the models trained on random subsets match the model trained on the full dataset

Inclusion / Exclusion Criteria

Data preparation

1. Download 1500 paper set
2. Install and run pre-rob on the papers
   (a) Get data from the "exclusion" column in the output spreadsheet. If the value for a given paper is > 0.5, consider it as a positive, otherwise a negative.
3. Repeat for Barzooka (`https://github.com/quest-bih/barzooka/commit/8ab662db3f2cc4b691f17bf508956952260d58c6`)
   (a) Extract results from the 7th output column, which is a binary indication if any flowchart was found.
4. Repeat for SciScore
   (a) Get the "Inclusion and Exclusion Criteria" field in the Rigor Table from the outputted JSON, and consider any extracted sentence as a positive, and a "not required" or "not detected" as a negative.
5. Aggregate data from the 3 tools into a spreadsheet with 2 columns
   (a) Take all papers where at least one tool disagreed on the binary presence of inclusion/exclusion criteria, and make a row for each
   (b) First column in spreadsheet contains a link to the paper
   (c) Second column contains a sentence extracted from a randomly selected tool. If it found inclusion/exclusion criteria, show the sentence, otherwise show no sentence. Do not show anything for Barzooka.

Curation

1. Add 3 additional columns
   (a) First column is a "Decision" column that can take on the value "yes", "no", or "complicated"
   (b) Second column and third columns contain notes
2. Conduct first pass on all rows
   (a) For each row, check if "inclusion" or "exclusion" are present in the extracted sentence and the sentence designates a group or subject that is included/excluded. If yes, mark the decision as "yes". If sentence mentions including/excluding an experimental variable that is not a subject, then mark the decision as "no". If sentence does not clearly fall into these categories, mark as "complicated". If there is no sentence associated with the row, skip.
3. Conduct second pass on rows marked "complicated" and those without sentences
   (a) For each row, open up linked paper and look for any sentence or diagram in the manuscript that would indicate inclusion/exclusion. If a clear sentence could be found, paste it into the document in the notes column. Using information from the full paper, make a decision on whether inclusion or exclusion was present (removing the "complicated" designation).

Blinding

Data preparation

1. Download 1500 paper set
2. Install and run pre-rob on the papers
   (a) Get data from the "blind" column in the output spreadsheet. If the value for a given paper is > 0.5, consider it as a positive, otherwise a negative.
3. Repeat for SciScore
   (a) Get the "Blinding" field in the Rigor Table from the outputted JSON, and consider any extracted sentence as a positive, and a "not required" or "not detected" as a negative.
4. Repeat for CONSORT-TM
   (a) Get data from the "BLINDING" column in the output spreadsheet. Consider a paper a positive if any sentence within that paper has "TRUE" in the "BLINDING" column.
5. Aggregate data from the 3 tools into a spreadsheet with 2 columns
   (a) Take all papers where at least one tool disagreed on the binary presence of blinding, and make a row for each
   (b) First column in spreadsheet contains a link to the paper
   (c) Second column contains a sentence extracted from a randomly selected tool. If it found blinding, show the sentence, otherwise show no sentence.

Randomization

Data preparation

1. Download 1500 paper set
2. Install and run pre-rob on the papers
   (a) Get data from the "random" column in the output spreadsheet. If the value for a given paper is > 0.5, consider it as a positive, otherwise a negative.
3. Repeat for SciScore
   (a) Get the "Randomization" field in the Rigor Table from the outputted JSON, and consider any extracted sentence as a positive, and a "not required" or "not detected" as a negative.
4. Repeat for CONSORT-TM
   (a) Get data from the "RANDOMIZATION" column in the output spreadsheet. Consider a paper a positive if any sentence within that paper has "TRUE" in the "RANDOMIZATION" column.
5. Aggregate data from the 3 tools into a spreadsheet with 2 columns
   (a) Take all papers where at least one tool disagreed on the binary presence of randomization, and make a row for each
   (b) First column in spreadsheet contains a link to the paper
   (c) Second column contains a sentence extracted from a randomly selected tool. If it found blinding, show the sentence, otherwise show no sentence.

Power calculations

Data preparation

1. Download 1500 paper set
2. Install and run SciScore on the papers
   (a) Get the "Power Analysis" field in the Rigor Table from the outputted JSON, and consider any extracted sentence as a positive, and a "not required" or "not detected" as a negative.
3. Repeat for CONSORT-TM
   (a) Get data from the "SAMPLE_SIZE_CALCULATION" column in the output spreadsheet. Consider a paper positive if any sentence within that paper has "TRUE" in the "SAMPLE_SIZE_CALCULATION" column.
4. Aggregate data from the 3 tools into a spreadsheet with 2 columns
   (a) Take all papers where at least one tool disagreed on the binary presence of power calculations, and make a row for each
   (b) First column in spreadsheet contains a link to the paper
   (c) Second column contains a sentence extracted from a randomly selected tool. If it found power calculations, show the sentence, otherwise show no sentence.

Open code detection

Data preparation

1. Download 1500 paper set
2. Install and run SciScore on the papers
   (a) Extract the "Code Information" from the SciScore Rigor table
3. Repeat for ODDPub
   (a) Get the column for open code in the output spreadsheet
4. Aggregate data from the 3 tools into a spreadsheet with 2 columns
   (a) Take all papers where at least one tool disagreed on the binary presence of open code, and make a row for each
   (b) First column in spreadsheet contains a link to the paper
   (c) Second column contains a sentence extracted from a randomly selected tool. If it found open code, show the sentence, otherwise show no sentence.

Software tools

1. Download 1500 paper set
2. Install and run SciScore on the papers
   (a) Extract the "Software Tools" objects from the Key Resources table
3. Repeat for SoftCite
4. Initial review decision column update:

   (a) Yes: If the entity is confirmed as software.
   (b) No: If the entity is not software.
   (c) Complicated: If the classification of the entity is ambiguous and needs further review.

5. Classification Criteria:

   (a) For URL Entities: Check if the URL is active and redirects to relevant software-related content.
   (b) For Non-URL Entities: Examine the presence of code, GitHub repositories, software versions, packages, and download options. Then confirm applications of software techniques or development practices.

6. Exclusion Criteria:

   (a) Entities solely related to databases, search functionalities, core facilities, organizational names, and hardware components without software or code integration.
   (b) Entities representing company names without direct involvement in software development or code provision.

7. Additional Columns:

   (a) RRID Presence: Indicate whether the entity has an RRID (Research Resource Identifier).
   (b) Notes: Provide detailed notes for entities marked as "Complicated," including the reason for ambiguity and any additional information relevant for the second review.

8. Second Review Pass:

   (a) Revisit "Complicated" entities as a group to resolve ambiguities and make final classifications.
   (b) Update the Decision Column based on collective assessments and additional information gathered.

Registration reporting

1. Download 1500 paper set
2. Install and run SciScore on the papers
   (a) Extract any mention results from the "Protocol Information" section in the Rigor Table
3. Install and run TRNscreener on the papers
   (a) Run file trial_identifier_search.R on the folder of full texts, and extract identifiers from the 3rd column of the output
4. Install and run ctregistries on the papers
5. Search for "NCT" in the paper full texts
   (a) No regex was used, only searching for the presence of "NCT"

Contaminated cell lines

1. Download 1500 paper set
2. Install and run SciScore on the papers
   (a) Extract the “Cell Line Authenticity” field from the Rigor Table
3. Repeat for PCLDetector

Abbreviations

XML - extensible markup language PMCID - PubMed Central ID RRID - research resource identifiers TP, TN, FP, FN - true positive, true negative, false positive, false negative

References

1. Vorland C, Allison DB, Brown AW. Semi-automated screening for improbable randomization in PDFs. In: Computational Research Integrity Conference.; 2021.
2. Horbach SP, Halffman W. The changing forms and expectations of peer review. Research integrity and peer review. 2018;3(1):8.
3. Oste DJ, Pathmendra P, Richardson RA, Johnson G, Ao Y, Arya MD, et al. Misspellings or “miscellings”—Non-verifiable and unknown cell lines in cancer research publications. International Journal of Cancer. 2024;155(7):1278–1289.
4. Babic Z, Capes-Davis A, Martone ME, Bairoch A, Ozyurt IB, Gillespie TH, et al. Incidences of problematic cell lines are lower in papers that use RRIDs to identify cell lines. Elife. 2019;8:e41676.
5. Barnett A. Automated detection of over-and under-dispersion in baseline tables in randomised controlled trials. F1000Research. 2023;11:783.
6. Carlisle JB. Data fabrication and other reasons for non-random sampling in 5087 randomised, controlled trials in anaesthetic and general medical journals. Anaesthesia. 2017;72(8):944–952.
